# Modeling Production-Living-Ecological Space for Chengdu, China: An Analytical Framework Based on Machine Learning with Automatic Parameterization of Environmental Elements

**DOI:** 10.3390/ijerph20053911

**Published:** 2023-02-22

**Authors:** Qi Cao, Junqing Tang, Yudie Huang, Manjiang Shi, Anton van Rompaey, Fengjue Huang

**Affiliations:** 1 Department of Civil Engineering and Architecture, Southwest University of Science and Technology, Mianyang 621000, China; 2 Geography and Tourism Research Group, Department of Earth and Environmental Sciences, KU Leuven, Celestijnenlaan 200E, 3001 Heverlee, Belgium; 3 School of Urban Planning and Design, Shenzhen Graduate School, Peking University, Shenzhen 518055, China; 4 Key Laboratory of Earth Surface System and Human-Earth Relations of Ministry of Natural Resources of China, Shenzhen Graduate School, Peking University, Shenzhen 518055, China

**Keywords:** production-living-ecological space (PLES), cellular automata, machine learning, scenario simulation, multi-objective dynamic weights

## Abstract

Cities worldwide are facing the dual pressures of growing population and land expansion, leading to the intensification of conflicts in urban productive-living-ecological spaces (PLES). Therefore, the question of “how to dynamically judge the different thresholds of different indicators of PLES” plays an indispensable role in the studies of the multi-scenario simulation of land space changes and needs to be tackled in an appropriate way, given that the process simulation of key elements that affect the evolution of urban systems is yet to achieve complete coupling with PLES utilization configuration schemes. In this paper, we developed a scenario simulation framework combining the dynamic coupling model of Bagging-Cellular Automata (Bagging-CA) to generate various environmental element configuration patterns for urban PLES development. The key merit of our analytical approach is that the weights of different key driving factors under different scenarios are obtained through the automatic parameterized adjustment process, and we enrich the study cases for the vast southwest region in China, which is beneficial for balanced development between eastern and western regions in the country. Finally, we simulate the PLES with the data of finer land use classification, combining a machine learning and multi-objective scenario. Automatic parameterization of environmental elements can help planners and stakeholders understand more comprehensively the complex land space changes caused by the uncertainty of space resources and environment changes, so as to formulate appropriate policies and effectively guide the implementation of land space planning. The multi-scenario simulation method developed in this study has offered new insights and high applicability to other regions for modeling PLES.

## 1. Introduction

The United Nations [1] predicts that the global urbanization rate will reach 72% by 2050. Such a large-scale urbanization process will lead to increasingly prominent conflicts, including development constraints, between production and living space [2,3,4], economic development and ecological protection [5], and urban expansion and cultivated protection [6,7,8,9]. To attain orderly development of space and promote balance and sustainability in production-living-ecological spaces (PLES), multiple stakeholders, including the public, scholars, and policy and decision-makers in multi-level authorities, have emphasized the spatial pattern of “promoting intensive and efficient production space, livable and moderate living space, and picturesque and beautiful ecological space” as planning for optimizing PLES [10,11,12,13].

Research on the PLES is regarded as an effective tool for managing land resources, coordinating the relationship between human and land resources, and promoting sustainable development [14]. By clarifying the control boundaries of urban, agricultural, and ecological space development, the quantity, structure, layout, and temporal changes of land space development and utilization can be determined [15]. However, how to maintain the PLES balance among the limited land resources in urban areas and promote sustainable development in terms of changing social and economic factors is one of the underdeveloped issues in the transformation of land spatial planning [16].

Machine learning is regarded as an effective tool to simulate PLES [17]. The core of its effectiveness is the suitability of input environment variables in multi-objective scenarios [16,18]. The research frontier is the dynamic automatic acquisition of multidimensional environment variables that influence relevant multi-object scenarios. However, there is no consensus in the academic community on how to conduct automatic acquisition [19,20], where scholars attempted diverse approaches to address this issue. In addition, when using machine learning under the frame of Cellular Automata (CA), two main classifications, urban land and non-urban land, are used in the simulation of urban land use, such as the delineation of urban growth boundaries (UGBs). Chengdu is one of the largest cities in western China and the core of the only urban agglomeration in Southwest China. Therefore, research on PLES in Chengdu is representative and valuable. 

The existing exploratory scenario modeling mainly achieves future scenario simulation by setting differentiated total space demand and emphasizes the current policy development orientation. However, it ignores the dynamics of the development of resource and environment elements in the multi-functional land space area in the scene [21]. The spatial simulation evolution mechanism of PLES with multi-objective scenario coordination needs to consider the aforementioned planning goals and index sets, such as food security, urban construction, and ecological protection, and determine the differentiated index values in the multi-objective scenario simulation model through the calculation of index thresholds [22], so as to study the system scheme that meets the multi-objectives of land space planning. Constrained by limited data, single consideration factors, and insufficient system cognition, the judgments of different index thresholds in previous planning systems were mostly combined with regional suitability analysis to calculate the range of the index [23,24].

The process simulation of key elements that affect the evolution of urban systems, such as population, activities, traffic, and land use, is still unable to achieve complete coupling, and there is a lack of a general and unified urban system model [25], studies on the organic integration of spatio-temporal differentiation lacking driving force and future land use scenario analysis [26]. Therefore, it is important to develop a multi-scenario spatial simulation model that takes into account different human activities within PLES and possesses high flexibility to meet the land-use allocation needs in the areas with complicated characteristics. So far, research on PLES in the western cities in China under multi-objective scenario simulation is still in the preliminary exploration stage, especially that based on multi-classification of land use type(s) and using artificial intelligent algorithms. 

Therefore, we aim to establish an analytical framework with multi-classification for scenario simulations of the future spatio-temporal pattern of PLES in Chengdu using machine learning methods: integrated bagging tree and change matrix of error gradient, followed by dynamic mining and update extraction of social and environmental driving forces affecting land spatial change under different target scenarios, and finally, the simulation of a multi-object scenario for land spatial change through “automatic index parameter matching”.

We constructed a multi-scenario simulation model of PLES based on the automatic parameterization process of system elements using the integrated learning classification model of integrated bagging tree (Bagging) and space-time cellular automata (CA) to mine the interaction between the system elements and the PLES.

The main contributions of this study can be summarized as follows.

- This study realizes the dynamic and automatic identification of the key elements that affect the evolution of the PLES under multi-object scenarios, which advances the toolbox for land use simulation methods and provide a framework for other case studies.
- Our investigation focuses on a typical southwestern Chinese city, which is one of the core cities in large urban agglomeration in southwest China. The evidence from this new case study could help mitigate the imbalance issue between research on the eastern and western regions in China and offer insights for other developing countries.
- We applied a finer land use classification data, combined with machine learning algorithms and multi-objective scenario simulation to study PLES, which offers new perspectives of land-use simulation and analytics for policy and decision makers.

## 2. A Brief Literature Review

### 2.1. The Core of PLES: Automatic Parameterization of Environmental Element

Previous studies have determined the threshold values of different indicators to calculate the range of indicators by combining regional suitability analysis [24,27], the entropy method [28], the analytic hierarchy process [29], and other mathematical statistics. Spatial models combined with artificial intelligence algorithms, such as the CLUE-S model, system dynamics (SD), and multi-objective programming based on RS and GIS, are widely used in land use optimization schemes to achieve coupled optimization of structure and layout [27,30,31,32]. The swarm intelligence optimization algorithm in the land use optimization configuration model is mainly based on adaptive rules and uses machine learning methods to simulate future multi-scenario land use configurations. Safitri et al. [33] used the support vector machine (SVM) to build a land suitability model in Java, Indonesia, to improve the accuracy of spatial allocation and evaluated the benefits of spatial allocation based on the hierarchy of physiological needs. Clarke et al. [34] refined the research patches and used the genetic algorithm (GA) to improve the efficiency of the CA prediction model and predict land use in California in 2100; Edan et al. [35] used CA and artificial neural network (ANN) algorithms to determine the land use change trend in eastern Iraq by 2030 and the impact of land use change on surface temperature. However, the automatic parameterization of environmental elements, whether using machine learning or other PLES research methods, is still under development.

### 2.2. Empirical Research on PLES

Research on PLES is relatively concentrated in developed cities, such as those in the southeastern regions of China, and mainly focuses on the following aspects: (1) Definition of the functional implications: For example, studies have discussed the three perspectives of function of land, landscape, and ecosystem services in Hangzhou [36,37]. (2) Identification and delineation: Some research prove that the PLES functional classification applies differently under different research scales and perspectives in Hebei province. The most common dentification methods can be roughly divided into two: the merge and classification method and the index quantitative measurement algorithm [38,39]. (3) Dynamically evolving features: Some studies analyze the change characteristics of PLES in chronological order, such as [40]; moreover, some scholars use land use dynamic change models to analyze the temporal and spatial evolution characteristics of PLES at different scales from different angles in the Fujian Delta urban agglomeration [41]. (4) PLES pattern simulation optimization: Much research has approached the optimization path and pattern reconstruction model of land space from the macro, meso, and micro scales, such as the optimized spatial allocation of construction land in Yangzhou and research on village-type identification based on the evolution simulation of PLES in Hunan, which has enriched the theory of land space optimization research [42,43]. However, insufficient attention has been paid to the western cities. In the Chinese context, the issue of east-west balance is very important. Therefore, we focus on the development of PLES in the western region.

### 2.3. PLES Combined with Multi-Categorical Land Use Data and Machine Learning Algorithms

Research on the optimal allocation of land is mainly concentrated on the evaluation of current resources and the environment, and the use of land units is examined using the multi-criteria decision-making model, supplemented by GIS *spatial* analysis. Chuvieco presented linear programming (LP) as a promising tool for spatial modeling within a GIS with four land types: forest land, rained cropland, irrigated cropland, and urban land [44]. Chen [45] developed an innovative method that is capable of simulating UGB alternatives with economic and ecological constraints based on patch-based cellular automaton in the Pearl River Delta (PRD), in southeastern China. The multi-scenario PLES simulation needs data yielding a finer classification of land use type to solve the problem of land use spatial layout optimization for food security, urban construction, and ecological protection and determine the differentiated index value in the multi-objective scenario simulation model through index threshold calculation [46]. However, current urban simulation approaches, such as the study of urban boundaries based on CA, mainly simulate two categories of land (urban land and non-urban land). In-depth research on the multi-objective scenario simulation of PLES using data on multi-category land cover/use combined with artificial intelligence algorithms is thus necessary.

Therefore, we focus on studying the PLES of typical big cities in western China and develop a scenario simulation framework combined with the Bagging-Cellular Automata (Bagging-CA) to generate various environmental element configuration patterns with more detailed land cover/use data classifications.

## 3. Data Collection

### 3.1. Study Area

**Figure 1:**
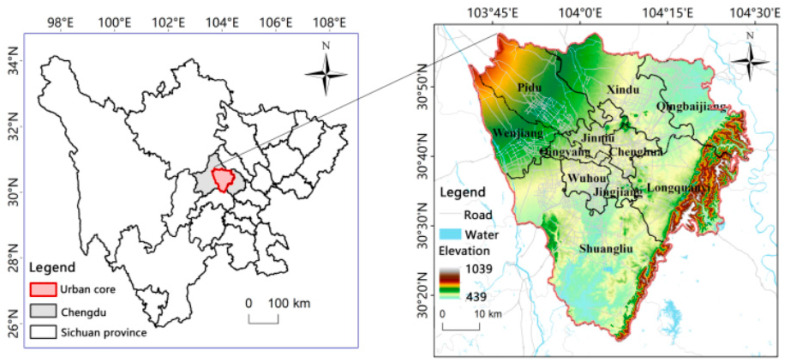
The location of the study area.

The case area in Chengdu, China, with geographical coordinates between 102°54′–104°53′ E and 30°05′–31°26′ N, is located in southwestern China and in the western Sichuan Basin. Chengdu covers an area of 14,335 square kilometers. As of the end of 2021, the permanent population 21.192 million, and the urbanization rate of the permanent population was 79.48%. It is the core city of the “Chengdu-Chongqing” city agglomeration in China, a high-tech industrial base, commercial logistics center, and comprehensive transportation hub. The overall terrain in the area is inclined from northwest to southeast, with abundant precipitation, vertical and horizontal river networks, rich products, and developed agriculture. The GDP was 1991.698 billion yuan (i.e., CNY) in 2021, which makes Chengdu the largest city in western China. This case area is mainly the urban core area of Chengdu, with a total area of 3680.04 km^2^ and an average elevation of 500 m, including 11 areas (Figure 1).

### 3.2. Data Source

The land use data (30 × 30 m^2^ resolution) for 2000, 2005, 2010, 2015, and 2020, and the Digital Elevation Model (DEM) were obtained from the Data Center for Resources and Environmental Sciences, Chinese Academy of Sciences (https://www.resdc.cn, accessed on 14 January 2023). The overall accuracy is 94.3% [47].

In this study, we use:

1. geological conditions, including geological disaster points and digital elevation model (DEM).
2. ecological environment conditions, including ecological environment quality, biodiversity, net primary production (NPP), farmland production potential, soil erosion, and normalized difference vegetation index (NDVI);
3. climatic conditions, including annual average temperature, annual precipitation;
4. economic conditions, including point of interest (POI) and night lights as the driving factors for the simulation model inputs.

These data were collected from the Data Center for Resources and Environmental Sciences of the Chinese Academy of Sciences (https://www.resdc.cn/, accessed on 14 January 2023). Vector data such as that on railways, roads, and settlements were drawn from OpenStreetMap (https://www.openstreetmap.org/, accessed on 8 January 2023). The data for population and GDP distribution, with a resolution of 1 km, were obtained from the Global Change Science Research Data Publishing System (http://www.geodoi.ac.cn/WebCn/CategoryList.aspx?categoryID=9, accessed on 14 January 2023). All of these data are freely accessible to the public. (See detail in Appendix A)

### 3.3. Data Treatment and Pre-Processing

In this study, we split the data into two subsets and used a five-fold cross-validation method to test the model performance. The data collected for the study area from 2010 to 2015 were used as the training set for model calibration and those from 2015 to 2020 as the testing set for model validation.

All these numerical variables were normalized to [0,1] using simple statistical normalization and resampled in GIS, with a spatial resolution of 1 km. Land use types were reclassified into (1) cultivated land, (2) woodland, (3) grassland, (4) water area, (5) urban land, (6) rural settlements, and (7) other construction land. All these land use data were converted into unit size of 100 × 100 m^2^, which is also the unit size of the subsequent implementation simulation, with a total of 369,768 units covering the whole study area.

The spatial variables were preprocessed by ArcGIS 10.7. The kernel density analysis was performed on POI, whereas the slope aspect analysis was performed on DEM. The neighbor analysis was then performed on distance factors. The variables of proximity to the town center/city center/river, terrain slope, and water quality conditions were kept constant, as these conditions were relatively stable during the study period.

## 4. Methods

### 4.1. Analytical Framework

**Figure 2:**
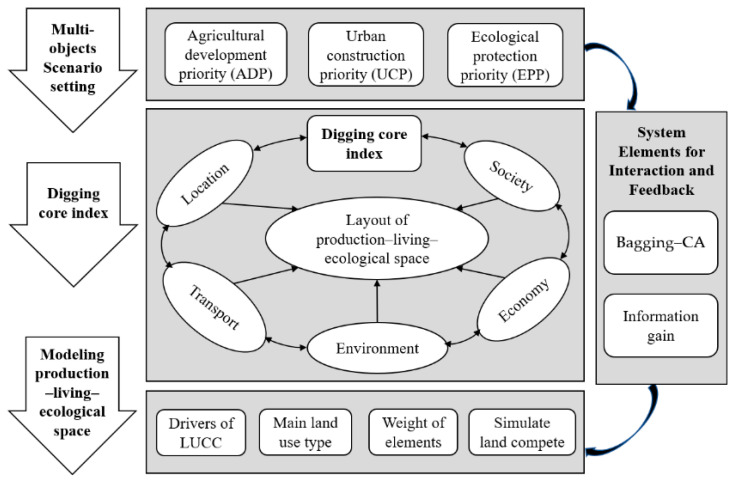
A sketch of the methodological framework.

To accurately describe the interactive feedback between the system of “economy-society-nature” and national land use space, the integrated learning classification model was used to mine the interaction between the system elements and the national land space. We then obtained the degree of influence (contribution) of each element index on various land use spaces by changing the number of model input elements to bring about gradient change in the classification prediction error to explore the key influencing parameters of the interaction (Figure 2).

Using the change matrix of the error gradient, we selected and reorganized the combination of key indicators for each type of spatial land use differentiation. In the CA conversion rule, only the conversion probability of the core land space set for each scenario was updated according to the difference of the core land space in each scenario. Through the above two steps, an automatic parameter adjustment process was constructed, and the CA scenario simulation results were optimized. Finally, the simulation of multi-scenario land space changes was realized by “automated index parameter matching”.

The training data for land use included those for the years 2000, 2005, 2010, 2015, and 2020, whereas the simulation year was 2025. To calibrate and check the usability of the model, the Kappa coefficient and the samples in the test set that were used to construct a mesh-by-grid confusion matrix between the simulation results and actual land use patterns. We then used the model to predict the changes in land use in 2025.

**Figure 3:**
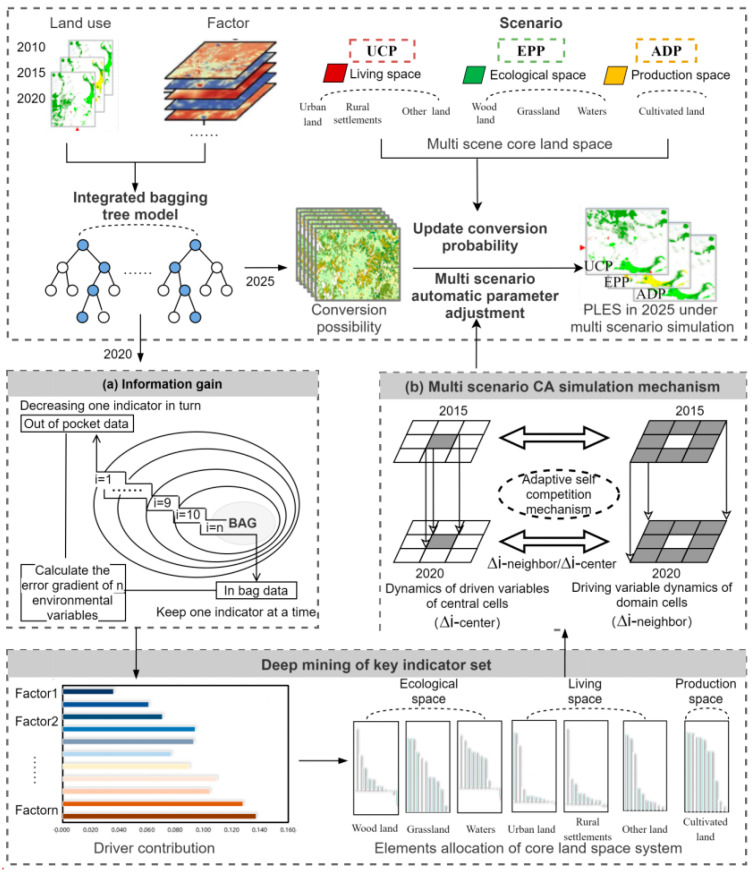
PLES simulation mechanism based on the automatic parameterization of environmental elements. (**a**) Using the change matrix of the error gradient, we selected and reorganized the combination of key indicators for each type of spatial land use differentiation; (**b**) In the CA conversion rule, only the conversion probability of the core land space set for each scenario was updated according to the difference of the core land space in each scenario.

We first sorted the interrelationships of elements in the multi-functional land use space system, identified the key influencing factors related to the spatial pattern of PLES, and established a many-to-many system relationship between PLES and the resource environment, thereby realizing the differentiated configuration of environmental elements under multiple scenarios. Then, by integrating the ensemble bagging tree model and the dynamic CA model, a multi-scenario PLES simulation mechanism based on “target-process-pattern” was constructed, as shown in Figure 3. First, the evolution characteristics of PLES were analyzed from the two aspects of total change and type transformation. Second, based on the “information gain” theory, the bagging algorithm was used to explore the key factors and driving mechanisms of PLES by examining the natural conditions, transportation location, socioeconomic level, and climate-environment changes and to build the parameter evaluation framework of urban construction, ecological protection, and agricultural production. Finally, the scenario was simulated and analyzed using the spatio-temporal CA model to evaluate the impact of the key indicator set on the path of territorial space demand in the urban core area of Chengdu in 2025, assuming the dynamic development of driving factors.

### 4.2. Simulation Scenario Design

To better promote coordinated development between agricultural development, urban construction, and ecological protection, this study defined three future land use demand scenarios, namely, (1) agricultural development priority (ADP), (2) urban construction priority (UCP), and (3) ecological protection priority (EPP) (Table 1).

The bagging model was used to complete the reorganization and configuration of key elements of the main functions of PLES. The spatio-temporal CA model was used to automatically read the recombination information of key elements of the main functional land of PLES, and the CA conversion rules were constructed according to the degree of change in the past five years. Then, according to the differentiation of the core land use space in each scenario, only the transformation probability of the core land use space set in each scenario was updated. In this process, the multi-scenario simulation mode was realized through automatic index parameter matching.

### 4.3. Bagging Algorithm

To accurately describe the degree of social environment development and its corresponding impact on land use, the contribution of key driving factors in multi-objective scenarios, that is, the weight of different indicators must be determined. To do so, we applied the bagging algorithm here. The bagging algorithm is a method of generating multiple base classifiers and using them to obtain aggregated predicted values [51]. It is a popular method of estimating standard errors and standard deviations and constructing parameter intervals [52]. In practical applications, the prediction of unbalanced sample data categories will increase the risk of misclassification of minority class samples into most samples, and the bagging algorithm has proved effective in solving such problems [53].

Based on the integrated bagging tree classification model, we used socioeconomic variables as input factors and 2020 land use types as output factors. In line with the information gain principle in information theory [54], this study explored the key parameters influencing the driving factors and obtained the impact degree (contribution degree) of each factor index on various land use spaces. Accordingly, the index weight value of the key influencing factors of the main function of PLES was established through the following implementation path:

1. First, the information entropy of N-dimensional features in the initial system was calculated.
2. Next, we removed the features sequentially and calculated the information entropy carried by the new system after removing each feature. Then, by calculating the difference between the information entropy carried by the initial system and that by the new system, the information gain of each feature on the whole data set was evaluated, and the estimation error of the out-of-pocket data was obtained.
3. The information entropy carried by the new system after only one feature was kept in turn was calculated. The difference between the information entropy carried by the new system and the average information entropy carried by each feature of the initial system was calculated, and the estimation error of the data in the bag was obtained.
4. The estimation errors of in-bag and out-of-bag data were averaged to obtain the degree of contribution of each feature to the whole data set, which was the weight of the corresponding index.

The above steps were implemented in the bagging classification model. Suppose a data set comprises N-dimensional features and C sample categories. The number of correct samples *N(j,j)* for each type of land use prediction was obtained by classification prediction after calculating N effective eigenvalues of the input layer, the number of samples *N(j,:)* for each type of land use was counted, and the information entropy of N-dimensional features in the initial system was calculated (Equation (1)).

(1) $$\overset{j=1}{\underset{j\in C}{\prod }}E_{j}=N_{\left(j,j\right)}/N_{\left(j,:\right)}$$

Then, after assigning a feature to 0 in turn, the remaining N−1 features retained their effective values; we calculated the number of samples *N1(j,j)* for each type of land use predicted by classification prediction after inputting N-dimensional features, counted the number of samples *N1(j,:)* for each type of land use, and calculated the information entropy of N-dimensional features in the new system (Equation (2)).

(2) $$\overset{i=1,j=1}{\underset{i\in N,j\in C}{\prod }}E1_{i,j}=N1_{\left(j,j\right)}^{i}/N1_{\left(j,:\right)}^{i}$$

Finally, only one valid feature was retained in turn, and the remaining *N-1* features were all assigned 0. The number of samples *N2(j,j)* for each type of land use predicted by the new system classification after the input of N-dimensional features was calculated. The number of samples *N2(j,:)* for each type of land use was counted, and the information entropy of N-dimensional features in the new system was calculated (Equation (3)).

(3) $$\overset{i=1,j=1}{\underset{i\in N,j\in C}{\prod }}E2_{i,j}=N2_{\left(j,j\right)}^{i}/N2_{\left(j,:\right)}^{i}$$

In the above equation, i represents participation in the operation of the new system after removing or retaining the $i_{th}$ eigenvalue each time, and j represents the $j_{th}$ type of land. *(j,j)* represents the samples with correct classification prediction, and *(j,:)* represents all the input samples of class j.

The information entropy (*E_j_*) carried out by the initial system was subtracted from the information entropy (*E1_i,j_*) carried by the new system after eliminating each feature, and the information gain value of the eliminated features (Equation (5)) was the contribution degree (*W1_i,j_*) of the out-of-pocket data to the whole data set.

(4) $$\overset{i=1,j=1}{\underset{i\in N,j\in C}{\prod }}W1_{i,j}=\left(E_{j}-E1_{i,j}\right)/\left(E_{j}\times \frac{C-1}{C}\right)$$

After retaining a feature, the information entropy ($\frac{E_{j}}{C\times N}$) carried by the average feature in the initial system was subtracted from the information entropy (*E2_i,j_*) carried by the new system to obtain the information gain value of retaining a feature (Equation (5)), which was the degree of contribution (*W2_i,j_*) of the data in the bag to the whole data set.

(5) $$\overset{i=1,j=1}{\underset{i\in N,j\in C}{\prod }}W2_{i,j}=\left(E2_{i,j}-\frac{E_{j}}{C\times N}\right)/\left(E_{j}\times \frac{C-1}{C}\right)$$

The degree of influence of each feature on each type of sample was obtained by the weighted average calculation of the degree of contribution of data outside and inside the bag, namely, the weight parameter (Equation (6)). In the above equation, $\frac{C-1}{C}$ represents the initial error coefficient of classification prediction.

(6) $$\overset{i=1,j=1}{\underset{i\in N,j\in C}{\prod }}W_{i,j}=\left(W1_{i,j}+W2_{i,j}\right)/2$$

The influence degree of all characteristics (social environment variables) on each type of land use space was ranked in descending order, the first six environmental variables with the greatest influence on the change of land use in each type of space were extracted, and the index parameter set of key driving factors of PLES planning was constructed (Table 2). This is used to develop the model of PLES under multi-objective scenarios based on spatio-temporal CA in Section 4.4. The contribution degree of the output environmental variables to the whole model system from high to low was as follows: biological richness (0.137), farmland productivity potential (0.128), GDP (0.109), ecological environmental quality (0.104), soil erosion (0.094), NDVI (0.093), night light (0.090), NPP (0.077), POP (0.071), and precipitation (0.061). The index with the lowest contribution was temperature (0.036), at only 0.036.

### 4.4. Spatio-Temporal Cellular Automata Model

To assess the impact of relevant environmental elements in the surrounding area on the region, we used the CA model to simulate geospatial space as a unit array, wherein the CA unit, such as an area in the land use system, is considered its state and that of its neighbors at any time and evolved according to a set of improved transition rules. The scenario-based framework developed in this study allows the assessment of the future potential land use trajectory of the study area based on a set of predefined assumptions about changes in key environmental factors: when changes in environmental factors have reached a certain threshold in the past five years. By determining the key driving factors and weights, the spatio-temporal CA conversion rules were constructed; the land use conversion process is described in detail later in the article.

First, taking the 3 × 3 moor field as a research unit, the change degree of key environmental factors of central and neighborhood cells in the last two years was calculated, respectively. The calculation equation is as follows:

(7) $$\overset{i=1,j=1}{\underset{i\in n,j\in M}{\prod }}\Delta x_{\left(i,j\right)}^{3\times 3}=x_{\left(i,j\right)}^{t}-x_{\left(i,j\right)}^{t-1}$$

(8) $$\overset{i=,j=1}{\underset{i\in n,j\in M}{\prod }}\Delta x_{center\left(i,j\right)}^{3\times 3}=x_{center\left(i,j\right)}^{t}-x_{center\left(i,j\right)}^{t-1}$$

where $\Delta x_{\left(i,j\right)}^{3\times 3}$ is the variation difference of the $i_{th}$ index specific to type j land use in each unit grid in the 3 × 3 field for many years, $x_{\left(i,j\right)}^{t-1}$ is the value of the $i_{th}$ index specific to type j land use in the previous year, and $x_{\left(i,j\right)}^{t}$ is the value of the $i_{th}$ index specific to type j land use in the current year. M and n represent the type and quantity of land spatial patterns, respectively, and t represents the statistical year. $\Delta x_{center\left(i,j\right)}^{3\times 3}$ is the variation difference of the $i_{th}$ index specific to type j land use in the central cellular unit within the 3 × 3 moor.

Second, the ratio of the dynamic difference between the domain cell and the central cell was calculated (Equation (9)), the number and proportion of the key indicators greater than or equal to the given threshold (5%) in the 3 × 3 field cells were counted (Equation (10)), and the weighted average was assigned to the central cell (Equation (12)); here, $\Omega_{center_{j}}^{3\times 3}$ is the transition probability of the central cell evolving to the $j_{th}$ land use. $W_{i}$ is calculated from Equation (6).

(9) $$\overset{i=1,j=1}{\underset{i\in n,j\in M}{\prod }}\tau_{\left(i,j\right)}^{3\times 3}=\frac{\Delta x_{\left(i,j\right)}^{3\times 3}}{\Delta x_{center\left(i,j\right)}^{3\times 3}}$$

(10) $$\prod_{i\in n,j\in M}^{i=1,j=1}K_{\left(i,j\right)}^{3\times 3}=\frac{1}{9}Number_{\tau_{\left(i,j\right)}\geq 5\%}^{3\times 3}$$

(11) $$\overset{j=1}{\underset{j\in M}{\prod }}\Omega_{center_{j}}^{3\times 3}=\frac{1}{i}\overset{i=1,j=1}{\underset{i\in n,j\in M}{\sum }}W_{i}K_{\left(i,j\right)}^{3\times 3}$$

Third, the initial land space conversion probability ($P_{j}$) predicted by ensemble learning was combined with the spatio-temporal CA simulation ($\Omega_{center_{j}}^{3\times 3}$) to estimate the combination probability ($PP_{j}$) of each cell grid (Equation (12)).

(12) $$PP_{j}=\overset{j=1}{\underset{j\in M}{\prod }}\left(\Omega_{center_{j}}^{3\times 3}+P_{j}\right)$$

Finally, we updated the initial probability value; the initial conversion probability ($P_{j}$) of the core land use space (Table 1) in each scenario was replaced by the combination probability ($PP_{j}$), while the initial conversion probability ($P_{j}$) of other land use spaces is retained. The final conversion probability of all types of land use under multiple scenarios was output, and the maximum conversion probability was the future land use type that was more likely to change (Equation (13)). $P_{FD}$*/*$P_{ED}$*/*$P_{UD}$ were simulated conversion probabilities under the three scenarios of priority: ADP, EPP, and UCP, respectively.

(13) $$Class=Argmax[\begin{array}{cc} P_{\mathrm{ADP}} & ①\\ P_{\mathrm{EPP}} & ②\\ P_{\mathrm{UCP}} & ③ \end{array}.$$

Based on the key indicator parameter configuration obtained in Section 3.3, the spatio-temporal dynamic CA model was applied to simulate three scenarios for the Chengdu urban core area in 2025. The spatial modeling of the three scenarios provided the basic assumptions associated with each scenario and differentiated patterns of land use spatial change. The ADP scenario showed substantial loss of natural land cover and contraction of rural settlements. The EPP scenario experienced a modest decline to a slight increase in natural land cover. The UCP scenario was characterized by substantial loss of natural land cover and expansion of urban land use.

## 5. Results

### 5.1. Model Performance

To quantitatively evaluate the simulation results, we used the mesh-by-grid confusion matrix (Table 3) and Kappa coefficient to verify the simulation results of the study area in 2020. For the accuracy evaluation results, the Kappa coefficient of 0.66 indicated that the prediction results are highly consistent with the actual situation.

**Figure 4:**
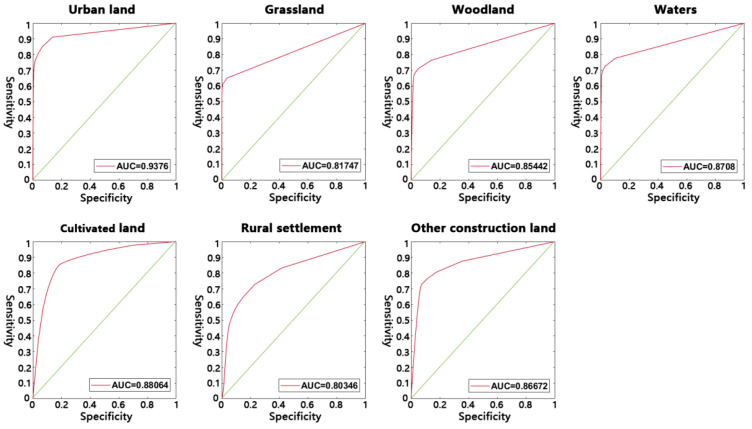
ROC curves and AUC values of each land use types fitted by the bagging model.

Furthermore, we also calculated the area under the curve (AUC) values for each land use type from their receiver operating characteristic (ROC) curves (Figure 4). We found that the AUC values of cultivated land, woodland, grassland, water area, rural settlement, and other construction land were all greater than 0.8, and the AUC value of urban land was greater than 0.9, which strongly suggest that the probability of occurrence suitable for each land use type can be well explained by the selected drivers.

We compared the simulated 2020 land use pattern with the actual land use pattern to measure the model’s performance. While a Kappa of 0.66 with an accuracy of 0.76 is not optimal, it can still be considered acceptable, compared to previous studies’ findings, for the period from 2015 to 2020 in the study area. For example, in the study by Liu et al. [47] the Kappa value of six-category land use dynamic modeling ranged from 0.71 to 0.79. Chen et al. [55] also reported a land use prediction Kappa of 0.65 to 0.83. Additionally, Chen et al. [56] obtained an accuracy range from 55.61% to 62.66% when comparing various prediction models, which was slightly lower than the 76% accuracy of our seven-classification model.

### 5.2. Analysis of the Evolution of the PLES

**Figure 5:**
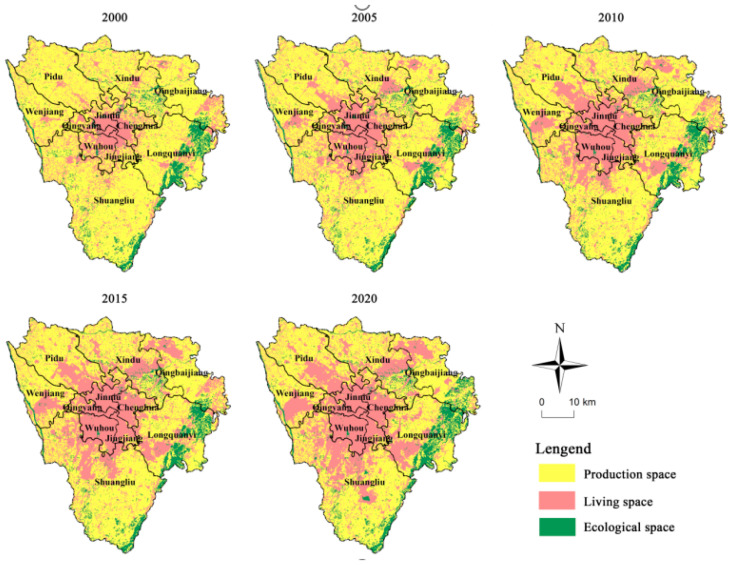
Spatial distribution pattern of PLES in Chengdu urban core area from 2000 to 2020.

To better observe the evolutionary trend of PLES in the study area, we analyzed it for the period of 2000–2020. As shown in Figure 5, the living spaces are primarily distributed in the central urban area during the study period, in the form of point-planes in each urban area, with significant spatial dispersion. The formation and distribution pattern of urban spaces emphasizes the influence of social economy, transportation location, and environment. Ecological space is mainly distributed around the ecological protection area, showing obvious linear characteristics, such as Longquanyi Mountains in the southwest and Minjiang River coastal areas in the southwest. The distribution characteristics of the ecological space are mainly determined by differences between natural attributes, such as terrain, climate, and the environment of different geographical units on one hand and social attributes such as human development intensity and environmental governance ability on the other hand. The distribution of production space is widespread and advantageous. The terrain in this area is flat and open, with a deep layer of soil, adequate light, and sufficient water conditions [57].

The dynamic degrees of production space, living space, and ecological space in the study area were 0.27 %, −0.08 %, and −0.73 %, respectively, during the period of 2000–2020 (see Table 4). This indicates that the change trend of PLES was mainly manifested as the stable growth of production space, a slight decrease in living space, and a significant decrease in ecological space. During the study period, the production space increased by 87.67 km^2^. The living space area decreased by 24.88 km^2^, in which the proportion of urban land increased from 7.27% to 16.92%, other construction land increased from 2.49% to 8.36%, and rural residential land decreased from 34.99% to 18.79%. The area of ecological space decreased by 62.79 km^2^, in which the proportion of forest area decreased from 7.34% to 7.15%, grassland decreased from 0.77% to 0.37%, and water area decreased from 3.73% to 2.23%.

### 5.3. Multi-Scenario Simulation for 2025

**Figure 6:**
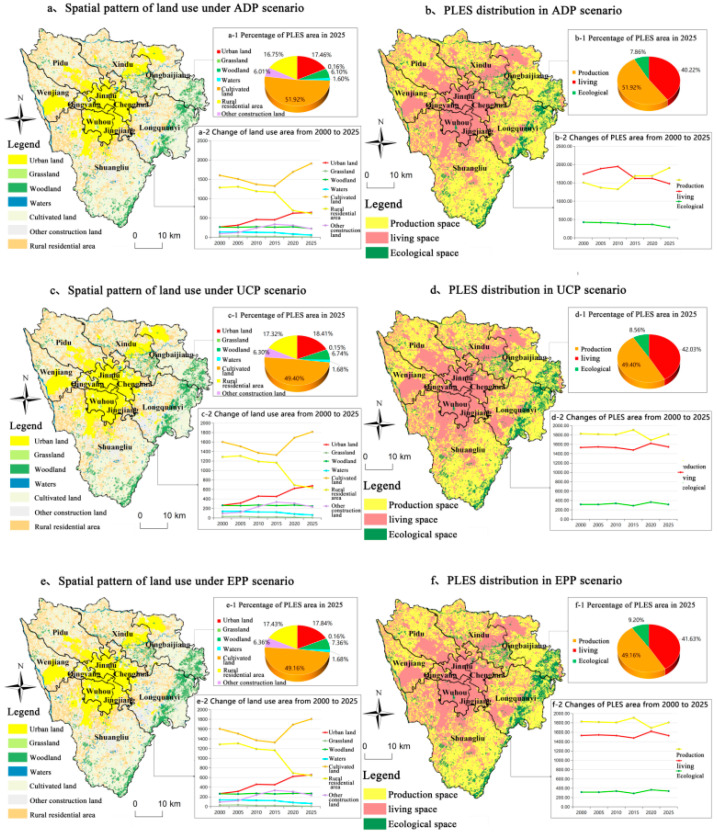
PLES pattern of Chengdu urban core area under different projected development scenarios in 2025. (**a**) shows the spatial layout of the seven types of land use under the agricultural development scenario. (**a-1**) shows the area percentage of the seven types of land use under the agricultural development scenario in 2025. (**a-2**) shows the change trend of the area of the seven types of land use under the agricultural development scenario from 2000 to 2025; (**b**) shows the spatial layout of the three types of space under the agricultural development scenario. (**b-1**) shows the area percentage of the three types of space under the agricultural development scenario in 2025. (**b-2**) shows the change trend of the area of the three types of space under the agricultural development scenario from 2000 to 2025; (**c**) shows the spatial layout of seven types of land use under urban construction scenario. (**c-1**) shows the area share of seven types of land use under urban development scenario in 2025. (**c-2**) shows the change trend of area of seven types of land use under urban construction scenario from 2000 to 2025; (**d**) shows the spatial layout of three types of space under urban construction scenario. (**d-1**) shows the area share of three types of spaces under the urban construction scenario in 2025. (**d-2**) shows the change trend of three types of spaces under the urban construction scenario from 2000 to 2025; (**e**) shows the spatial layout of seven types of land use under the ecological protection scenario. (**e-1**) shows the area share of seven types of land use under the ecological protection scenario in 2025. (**e-2**) shows the change trend of seven types of land use under the ecological protection scenario from 2000 to 2025; (**f**) shows the spatial layout of three types of space under the ecological protection scenario. (**f-1**) shows the area share of three types of space under the ecological protection scenario in 2025. (**f-2**) shows the area change trend of three types of space under the ecological protection scenario from 2000 to 2025.

Finally, we used the model to predict how the land use pattern would alter in 2025. The internal land use of living space is mainly reflected in the continuous expansion of urban land, which is mainly located around the current built-up area, presenting a spatial distribution pattern of one core and multiple centers. The results (Figure 6) showed that compared to 2020, the living space area of the three modes would have reduced significantly and the ecological space would have continued to decrease, whereas the production space would have significantly increased. In terms of the area ratio of PLES space, production space would have accounted for the largest proportion in all scenarios.

The overall scale of rural settlements would have continued to shrink, distributed in the periphery of urban land. Other construction land would have been constantly exported. The internal land use of ecological space would have been mainly characterized by the continuous sharp decrease in grassland area, which would have been identified as the outer ring area of the main city (i.e., the large parks around the city would have been identified as artificial grasslands). In the case of woodlands, scenarios of expansion and contraction would have varied, but with a primary concentration in the southeast Longquanyi Mountains. The water would have been constantly pumped out. The internal land use of production space would have mainly manifested as the increase in cultivated land.

In the ADP model, the growth of metropolitan areas in the scenario would have been somewhat suppressed. Strict cultivated land protection measures would not only have limited the number of future urban areas—which would have allowed these scenarios to develop in the least urban area—but also guided the direction of urban development. A large number of small woodlands surrounding the city would have transformed into agricultural land under this development mode. The more obvious change would be in regions that are far from cities, where a large number of rural settlements would be degraded to agricultural land. In addition, in this scenario, large areas of woodland would be swallowed up by agricultural land in areas far from cities.

In the UCP model, the other construction land near urban areas can be easily reused and developed into urban land, which would be prominent in Longquanyi and Shuangliu. This means that Shuangliu and Longquanyi would have more development opportunities than other administrative regions under this development model. In addition, the urban sprawl of surrounding towns would be connected to the metropolitan area by eroding cultivated, small woodland, and rural settlements on the edge. However, small woodlands and those cultivated on the fringes of cities would be significant for ecosystem functions. Thus, the compactness of the urban form would come at the expense of the quality of the urban environment.

In the EPP model, most of the cultivated land would be converted to woodland on the east side, which would be also the biggest difference between the UCP and ADP scenarios. However, for grasslands with the same ecological value, the reduction would still continue in this scenario. Therefore, attention needs to be paid to grassland restoration on other construction land in the suburban areas.

## 6. Discussion

### 6.1. The General Law of Scenario Evolution

The simulation results under the agricultural protection scenario can fully protect the cultivated land resources and maintain food security. The production space in all scenarios showed that the total amount of cultivated land would continue to increase, especially in the ADP scenario. The results of this study thus indicate that the reduction in future production space has been effectively controlled, and ecological space is mainly threatened by the development of production space. Moreover, ecological space has become the main source of the expansion of living space. This finding is also supported by previous evidence from Li et al. [8]. However, our research results further confirm that the mutual restriction between production space and living space is not only regarding competition for ecological space but also the annexation of rural settlements within the living space. On the one hand, with the intensification of urbanization, cities continue to absorb rural population) [21]. On the other hand, with the emergence of the “hollow villages”, a kind of imbalance between the transfer of rural population and the shrinking size of residential bases, and the vicious circle of rural development, rural sustainable development inevitably requires rural transformation development [58,59].

The simulation results in the context of urban construction can meet the demands of social and economic development for construction land. Urban land growth under this scenario would be higher than that under the other two scenarios, which is consistent with the research results of Cao et al. [60]. However, there is a difference in the extent of urban land growth, mainly because of the time difference between the studies. The model established in this study only simulated the spatial change of land use in the urban core area of Chengdu from 2020 to 2025. Urban expansion is a gradual process, and it is not clear whether the city scale will expand substantially in such a short time [61]. However, it should be noted that in this case, living space gradually encroaches on ecological space from the periphery, and urban construction land and rural residential land tend to be intensively used. Such a result was also confirmed in the study by Zhao et al. [62]. In this scenario, there is a research consensus that the guaranteed area of construction land is the most important, the food demand and security of cultivated land are the main criteria, and the preservation of woodland, grassland, and water area—the minimum demands of human life and the environment—are the baseline; even this will impose a great burden on regional land resources. As cities continue to expand, concerns arise about their negative impact on the environment; however, this neglects the fact that urbanization may also have a positive impact on ecosystem restoration through population migration, advanced agricultural techniques, cleaner production strategies, and increased investments in ecological conservation. Relevant evidence on the positive effects of urbanization has been provided in previous case studies [63,64], although these findings may depend on a particular development stage, region, and size. Some also argue that although efficiency gains can be achieved, these gains may not be sufficient to offset overall resource requirements because of the rapid growth of urban systems [65]. In conclusion, the negative and positive impacts of urbanization on ecosystem services are indeed two sides of the same coin. The implications for other raising developing countries are that multi-functional land use patterns can be optimally allocated in space and time, which is extremely useful for coordinating stakeholder participation and addressing conflicts of interest in land use behaviors; this helps to promote high-quality utilization of land resources and balancing regional development and ecological protection [66].

The simulation results under the ecological protection scenario can effectively guarantee the ecological constrains. In this context, our results show that there are scattered ecological spaces around the urban space, which is called the “urban-agricultural space” in the study by Baró et al. [67]. These spatial distribution patterns can effectively reduce the pressure of human activities on limited natural resources, provide a transitional zone between urban and agricultural spaces, prevent agricultural space from being occupied, and limit soil and water pollution caused by various urban pollutants [68]. In the process of urban expansion, the transformation of natural land into artificial land, such as parks, gardens, and sports areas [69], will inevitably accelerate, which may change or destroy the function of natural ecosystems. However, in the context of urban expansion, the greatest protection for the ecological environment under the EPP scenario is to ensure to the greatest extent possible that the woodland and artificial grassland areas are not reduced—especially, in our case, with regard to the protection of woodland in the Longquanyi Mountains and small suburban grasslands. Furthermore, grassland restoration should be carried out on other construction land in the suburbs. This spatial layout can control the transfer of urban and rural land, improve the quality of the urban ecological environment, and effectively relieve the pressure of residential space crowding. In addition, the impact of urban growth on ecosystem functions varies with the spatial layout and configuration of urban land [70]. Although there is no consensus on what urban forms are sustainable, fragmented urban forms and their corresponding lifestyles tend to put greater pressure on ecosystem functions [71,72].

The results imply that the multi-scenario simulation approach in this study has the potential to be applied more widely in other areas, providing new insights for planners and decision-makers in long-term land use planning. The following key insights emerge: (1) The uncertainty of future land spatial changes requires the use of a multi-scenario model in prediction research. (2) Multi-scenario simulation can improve simulation authenticity through the automatic configuration of the weight parameters of key environmental factors. (3) Land spatial planning requires us to focus on regional differences and the dynamic development of regional environmental factors.

### 6.2. Discussion of the Multi-Scenario Simulation Model

Our prediction results are based on the land use change rules in the study area from 2000 to 2020 and fully consider the dynamic changes in the area’s social, economic, and environmental characteristics from 2015 to 2020. The framework that we have developed allowed us to produce PLES projections for multiple scenarios in the urban core area, which can be used to support territorial spatial planning and decision-making. The value of future projections is not pure prediction but for our ability to examine land use impacts across a range of potential future economic, ecological, and environmental changes with regard to biodiversity, water cycles, and climate adaptation and mitigation [73]. Estimates of future land use change constitute an important input to carbon climate projections [74], which in turn can be used to assess the consequences of potential greenhouse gas emissions and predict future climate change [75], while the simulation results can also be used to provide ex ante assessments of policies or as an early warning system for environmental impacts [76,77]. Prediction and estimation of greenhouse gas emissions and predicting future climate change are imperative to avoid consequences regarding the environment, production system, and health [78,79,80].

However, predicting PLES changes remains incredibly challenging and uncertain [81]. The uncertainty of many factors (such as environment and chance) may lead to inconsistencies between actual and predicted results [82,83] and will produce great changes in the development trend of land use space; therefore, it is necessary to consider uncertainty in the prediction. In PLES prediction, the spatial changes of the seven land use types under the three different scenarios were simulated by weighing the differences in the spatial functions of different land uses. However, owing to the future uncertainty of many factors, the amount of land space cannot be regarded as an inherent value; nevertheless, it can provide a reference value for the development of regional land use.

The current international consensus is that the contradiction between farmland protection and ecological conservation demands a better trade-off, and that more attention should be paid to avoiding the occupation of basic farmland in ecological restoration [84,85]. This study can serve as a reference for other cities and even countries regarding the dominant function and functional positioning of PLES, which helps ensure flexible development strategies for spatial planning.

To sum up, the multi-objective land space simulation model is an effective tool for analyzing the causes and consequences of land space changes under the conditions of future multi-scenario development related to socioeconomic and natural environmental driving forces. Our model can provide planners and researchers with effective simulation methods, help decision-makers formulate appropriate policies, and effectively guide the implementation of land and space planning.

However, we recognize that many factors may have limited the usefulness of our framework in other applications. The framework relies heavily on spatially explicit biophysical and socioeconomic data, and the model is parameterized (e.g., the parameterization of plaque characteristics based on historical land use data). Despite the model’s improvement upon the traditional implementation of scenario simulation, we could not exclude the possibility that we had missed other relevant explanatory variables. Different research fields and input data may also affect model performance. Future studies should consider additional economic and social factors such as income levels, employment rates, and accessibility [86], which could greatly improve model performance.

## 7. Conclusions

This study aimed to better inform urban planners and policy makers for sustainable urban development planning and explore possible future development paths, as well as “what-if” scenarios, which was achieved by identifying dynamic changes in the key factors influencing the primary functional use of PLES. To do so, we constructed a model to investigate the dynamic weight of the social-environmental driving factors that affected changes in the land space and established a system network relationship between the land space pattern and the multi-variable resources and environment under multiple scenarios. Eventually, we effectively predicted the changing trend of land space in this case study and offered several useful implications. This study yields the following main remarks:

(1) Multi-objective simulation models are effective tools for analyzing the causes and consequences of PLES changes under future multi-scenario development conditions related to dynamic changes in drivers. Complex linkage and feedback structures need to be understood to simulate multiple land use conversions under uncertain future conditions.

(2) This study realizes the dynamic and automatic identification of the key elements (automatic parameterization of environmental elements) that can help planners and stakeholders understand more comprehensively the complex land space changes caused by the uncertainty of space resources and environment changes, so as to formulate appropriate policies and effectively guide the implementation of land space planning.

(3) In this study, the change matrix of the error gradient was used to realize the dynamic mining and updating extraction of the social-environmental drivers of PLES change under different target scenarios. Several solutions were proposed to simulate the spatial trajectory of PLES in the scenario of human activities and natural evolution, which compensates for the defects of traditional scenario simulation, which make it necessary to set the future total amount of various types of land under multiple different scenarios to realize the maximum-probability simulation of spatial distribution of various land types in the future.

For future works, the proposed analytical framework could be tested in other case studies with more geographical and socioeconomic characteristics.

## Figures and Tables

**Table 1:** Multi-scenario setting rule description.

Scenario	Instructions	Core Land Space	Target	Reference
Scenario 1: Agricultural development priority (ADP)	Give maximum protection to arable land and strictly control the conversion of basic cultivated to other types of land.	Production space Cultivated land	Controls the quality and quantity of cultivated to ensure food security.	[48]
Scenario 2: Urban construction priority (UCP)	Make full use of living space to maximize the economic benefits of scale.	Living space Urban land; Rural settlements; Other construction land	Reveals the core driving force and potential threat to social stability and ecological environment under the priority city development mode.	[49]
Scenario 3: Ecological protection priority (EPP)	Set the maximum ecological space capacity to ensure the maximum ecological benefits provided by land use.	Ecological space Woodland; Grassland; Water area	Provides reference value for the delineation of ecological red line and promotes high-quality urban development.	[50]

**Table 2:** Key index parameters of core land use space under multiple scenarios.

Sce 1	Living Space						Sce 2	Ecological Space						Sce 3	Production Space	
	Urban Land	W	Other Construction Land	W	Rural Settlements	W		Wood Land	W	Grassland	W	Water Area	W		Cultivated Land	W
UCP	Farmland productivity potential	0.128	Farmland productivity potential	0.128	Biological richness	0.137	EPP	Biological richness	0.137	Farmland productivity potential	0.128	Night light	0.090	ADP	NDVI	0.093
	Ecological environmental quality	0.104	Soil erosion	0.094	Night light	0.090		GDP	0.109	GDP	0.109	Ecological environmental quality	0.104		Temperature	0.036
	Biological richness	0.137	Precipitation	0.061	Ecological environmental quality	0.104		Farmland productivity potential	0.128	NDVI	0.093	NDVI	0.093		POP	0.071
	GDP	0.109	GDP	0.109	NPP	0.077		POP	0.071	POP	0.071	Farmland productivity potential	0.128		NPP	0.077
	Night light	0.090	NDVI	0.093	POP	0.071		Soil erosion	0.094	Ecological environmental quality	0.104	GDP	0.109		GDP	0.109
	NPP	0.077	Biological richness	0.137	GDP	0.109		NPP	0.077	Night light	0.090	Soil erosion	0.094		Night light	0.090

UCP: urban construction priority; EPP: ecological protection priority; ADP: agricultural development priority; W: weights.

**Table 3:** Confusion matrix of the predicted versus actual land use patterns in 2020.

Land Use Types	Actual Land Use in 2020							
	Urban Land	Grassland	Woodland	Water Area	Cultivated Land	Other Construction Land	Rural Settlements	Total
Urban land	42,731	323	249	282	3970	3914	4455	55,924
Grassland	111	685	70	16	146	71	165	1264
Woodland	108	11	15,966	111	4417	3509	506	24,628
Water area	194	4	159	4895	1666	200	447	7565
Cultivated land	1675	21	3679	1107	130,503	5269	11,153	153,407
Other construction land	1950	33	628	297	9699	12,856	2227	27,690
Rural settlements	890	22	573	242	13,111	2472	45,013	62,323

Kappa Coefficient = 0.66, Overall Accuracy = 0.76.

**Table 4:** Evolution of PLES from 2000 to 2020.

Territorial Space Structure	Area (km^2^)					Dynamic Degree (%)				
	2000	2005	2010	2015	2020	2000 –2005	2005 –2010	2010 –2015	2015 –2020	2000 –2020
Production space	1602.20	1508.74	1369.90	1325.64	1689.87	−1.17%	−1.84%	−0.65%	5.50%	0.27%
Living space	1644.85	1739.98	1888.53	1948.17	1619.97	1.16%	1.71%	0.63%	−3.37%	−0.08%
Ecological space	428.28	426.62	416.91	401.54	365.50	−0.08%	−0.46%	−0.74%	−1.80%	−0.73%

## Data Availability

Data sharing not applicable.

## Appendix A

**Table A1:** Data source and processing.

Primary Data Source	Data Classification	Data Processing and Usage	Year	Data Download
Geographical conditions	Geological disasters	Nuclear Density Analysis	2020	National Earth System Science Data Center
	DEM	Slope and Aspect Analysis	2010	Geospatial Data Cloud
Ecological environment	Ecological environmental quality	Extract Value to Point; Extract the ecological and environmental quality conditions of sample units	2010/2015	Resource and Environmental Sciences Data Center
	Biodiversity		2000/2005	
	NPP		2010/2020	
	Farmland potential productivity		2000/2010	
	Soil erosion		2010/2015	
	Soil quality		2015	
	Water quality		2015	https://data.epmap.org/product/province_water (accessed on 14 January 2023)
	NDVI		2015/2020	Geospatial Data Cloud
Climatic conditions	Temperature	Extract Value to Point; Extract climate change from sample units	2010/2015/2020	Resource and Environmental Sciences Data Center
	Precipitation		2010/2015/2020	
Built environment	POI	Nuclear Density Analysis; Extract the urban development and construction conditions	2015/2020	http://59.175.109.173:8888/index.html (accessed on 14 January 2023)
	Nighttime light	Extract Value to Point; Extract the degree of economic development of sample unit	2020	https://www.arcgis.com/home/search.html?q=POI (accessed on 14 January 2023)
	Population density		2010/2015/2020	http://www.geodoi.ac.cn/WebCn/doi.aspx?Id=131 (accessed on 14 January 2023)
	GDP		2010/2015	http://www.geodoi.ac.cn/WebCn/doi.aspx?Id=125 (accessed on 14 January 2023)
	Distance factor	Neighbor Analysis; Extract the location advantage condition of the sample unit	2010/2015/2020	Resource and Environmental Sciences Data Center
				OpenStreetMap (http://www.openstreetmap.org/ (accessed on 14 January 2023))
	Road integration	Space Syntax; Extracting the road accessibility of sample units	2010/2015/2020	
Spatial location	Longitude	Extract the spatial location relationship of sample units	2020	Resource and Environmental Sciences Data Center
	Latitude			
Land use	Land use in historical period	Extract the historical land type of the sample unit	2000/2005/2010/2015/2020	
